# COVID-19 precautionary practices and associated factors among clients visiting a tertiary hospital, Addis Ababa, Ethiopia

**DOI:** 10.1371/journal.pone.0267000

**Published:** 2022-04-18

**Authors:** Hailemichael Bizuneh, Shikur Mohammed, Aman Yesuf

**Affiliations:** Epidemiology and Biostatistics Department, School of Public Health, Saint Paul’s Hospital Millennium Medical College, Addis Ababa, Ethiopia; Institute for Advanced Sustainability Studies, GERMANY

## Abstract

**Background:**

Despite efforts to contain the spread of COVID-19, Addis Ababa, the country’s COVID-19 epicenter, is experiencing a sharp increase in the number of cases and death rate. While poor public adherence to COVID-19 precautionary practices is evident, factors associated with it are not well studied. We aimed to assess the level of practice of COVID-19 precaution and associated factors.

**Methods:**

This was a hospital-based cross-sectional study conducted from February 1^st^ to 15^th^, 2021 at Saint Paul’s Hospital Millennium Medical College, a tertiary teaching hospital in Addis Ababa, Ethiopia. We used a structured questionnaire to conduct a face-to-face exit interview with clients visiting the hospital. Systematic random sampling was employed to recruit study participants. Binary and multivariable logistic regressions were implemented to examine factors associated with precautionary practices. Statistical significance was declared at p-value <0.05. The Crude odds ratio (COR) and Adjusted odds ratio (AOR) were reported with a 95% confidence interval.

**Results:**

We analyzed data obtained from 262 participants. The mean age of participants was 36 (SD+12) years. The majority (207, 79%) of the study participants had a favorable attitude towards prevention and control measures of COVID-19. A little over half (116, 55.7%) of the respondents had a satisfactory level of practice of COVID-19 precautions. Living in an area with strict enforcement of COVID-19 precautionary measures [AOR: 2.25, 95% CI (1.22–4.15)], and having a favorable attitude of COVID-19 prevention [AOR: 4.88, 95% CI (2.08–11.68)] were significantly associated with satisfactory COVID-19 precautionary practices.

**Conclusions:**

The level of practice of COVID-19 precaution was unsatisfactory. Favorable attitude and stricter enforcement of COVID-19 preventive measures might have contributed more to adherence to precautionary practices. The findings highlight the need for a public health education strategy targeted at improving attitudes of the community on COVID-19 focusing on the effectiveness of preventive measures.

## Introduction

Since its emergence in December 2019, the coronavirus disease (COVID-19) caused by severe acute respiratory syndrome coronavirus 2 (SARS-CoV-2) has progressed into a pandemic [1, 2]. Globally, as of May 11, 2021, over 158 million confirmed cases including over 3 million deaths were reported in Africa [3]. The second-most populous African country, Ethiopia, has reported over 260,000 confirmed cases and 3,888 deaths [4, 5]. Addis Ababa, the capital city, reported a sharp rise in positivity rate, health facility admissions, and deaths indicating a high community transmission [4]. In the past year, the Ethiopian Ministry of Health (MoH) has implemented comprehensive COVID-19 prevention measures. Notably, the ministry enacted a directive aimed to enforce COVID-19 Public Health and Social Measures (PHSM). Despite this effort, poor public adherence to precautionary measures seems to be a key factor in impeding efforts to slow down the spread of COVID-19 [6]. With only one percent of the country’s population vaccinated as of May 11, 2021, and a growing concern of new COVID-19 variants, proper practice of precautionary measures remains the first line of defense against COVID-19 [7].

According to the World Health Organization (WHO), engaging in the proper practice of precautionary behaviors including personal hygiene, wearing a face mask, and maintaining social distance, significantly contribute to controlling the spread of COVID-19 [6, 8]. A multitude of factors affect the level of individual prevention practices in a population. Among others, individual awareness, knowledge, attitude, and perception of risk are key in the adoption of precautionary practices [6, 8, 9]. However, evidence is scarce as to which of these factors contribute more to a practical adoption and adherence to such precautions. The few studies conducted previously were limited to subsets of the population, particularly health care workers [10–12]. This study aimed to investigate the level and factors associated with COVID-19 prevention practices among clients visiting a tertiary hospital in Addis Ababa.

## Methods

### Study design and setting

This hospital-based cross-sectional study was conducted from February 1^st^ to 15^th^ 2021 at Saint Paul’s Hospital Millennium Medical College (SPHMMC) in Addis Ababa, Ethiopia. This tertiary hospital is the second-largest referral and teaching hospital in the country. Currently, it has an inpatient capacity of 700 beds and an average of 1,200 emergency and outpatient clients daily. The hospital is one of the few COVID-19 centers in the capital city and currently runs the largest COVID-19 care center in the country.

### Study population and sampling

All clients visiting the hospital were the source population for the study, of which randomly selected clients were interviewed. Being an adult (>18 years) was an inclusion criterion. A single population proportion formula (n = [(Zα/2)^2^P(1-P)]/d^2^) with a marginal error of 5% (d = 0.05); and a standard score corresponding to 95% confidence (CI) (Z_*a*/2_ = 1.96) were considered to determine the sample size. We took the prevalence (p) of the practice of ‘hand washing’ from a similar study (77.3%) [13] which yielded the maximum sample size of 270. After considering a 5% non-response rate, the final sample size for this study was determined to be 284 clients.

We used a systematic random sampling technique to identify respondents and conduct a face-to-face exit interview. The number of patients who visited the hospital’s outpatient department on a daily basis three months before data collection was determined by consulting client register books. A sampling interval was determined by dividing the expected number of clients by the total sample size, which was then proportionally allocated to major departments in the hospital.

### Study variables and measurement

The dependent variable was level of COVID-19 precaution practices and the independent variables were classified into socio-demographic characteristics: age, gender, educational status, family size, marital status, and occupational status; knowledge level and source of information on COVID-19 transmission, prevention, and control methods; attitude towards COVID-19 prevention, and control measures; perceived level of risk of contracting COVID-19 and presence of underlying chronic illnesses of the respondent or their family.

We adapted Bloom’s cut-off point of >80% on aggregate score to rank respondents’ overall knowledge, attitude, and practice scores [14]. Practice, knowledge, and attitude were operationally defined as follows: Satisfactory practice: a score of > = 80% on nine items with a possible response and point score of “Always = 2”, “Occasionally = 1” and “Never = 0” assessing the practice of COVID-19 precautions. Adequate Knowledge: a score of > = 80% on 19 items with a possible response and point score of “Yes = 1” or “No = 0” assessing the knowledge of COVID-19 prevention measures, transmission ways, and disease symptoms. Favorable attitude: a score of > = 80% on five, five point-Likert scale items with a response and point score of “Strongly agree = 5”, “Agree = 4”, “Neutral = 3”, “Disagree = 1” to “Strongly disagree = 0” assessing the attitude towards COVID-19 prevention and control measures.

### Data collection

Data were collected by trained data collectors using a structured tool adapted from WHO’s COVID-19 knowledge, risk perceptions, precautionary behaviors survey tool [15]. The tool was pre-tested on a sample of clients at a nearby health center. The questionnaire was translated into Amharic (the local working language) and back to English to assess the consistency in the meaning of items in the two tools. The Amharic version of the questionnaire was used to interview respondents.

### Data analysis

We entered the data into Epi Info Version 7 and exported it to SPSS version 23 for analysis. Data were checked for outliers, cleaned and negatively worded items were reverse-scored and recoded. Descriptive statistics such as frequency, proportion, mean and standard deviation were computed. Binary logistic regression analysis was implemented to examine factors associated with COVID-19 precautionary practices. Variables with a p-value of 0.25 or lower on binary analysis were further analyzed in a backward stepwise multivariable logistic regression model. The model appropriateness assumptions were fitted to the data. Model fitness was checked using the Hosmer-Lemeshow test (P>0.05). A p-value of <0.05 and an Adjusted odds ratio (AOR) with a 95% Confidence Interval (CI) were used to report significant associations.

### Ethics statement

We obtained ethical clearance from SPHMMC Institutional Review Board. The participants were informed about the purpose of the study, confidentiality, voluntary participation and anonymity. Oral informed consent was obtained from all the respondents.

## Results

### Socio-demographic characteristics

A total of 284 clients were initially approached for the face-to-face interview. After excluding those who refused to participate and those with incomplete responses, 262 (92.2%) respondents’ data were included in the final analysis. The mean age of participants was 36 (SD+12) years. More than a third of the respondents (75.5%) had an educational level of secondary school and above. Table 1.

**Table 1:** Socio-demographic characteristics of respondents.

Characteristics	Frequency	Percentage
**Age**		
18–29	86	32.8
30–49	131	50
50–70	45	17.2
**Sex**		
Male	140	53.4
Female	122	46.6
**Religion**		
Orthodox	141	53.9
Muslim	60	22.9
Protestant or Jehovah’s witness	61	23.2
**Marital Status**		
Single	91	34.7
Married	156	59.5
Divorced, Widowed or Separated	15	5.8
**Educational status**		
Unable to read and write	17	6.5
Able to read and write	12	4.6
Primary (1-8^th^ grade)	35	13.4
Secondary (9-12^th^ grade)	90	34.3
College and above	108	41.2
**Occupational status**		
Employed	252	96.2
Unemployed	10	3.8
**Place of residence**		
Addis Ababa (capital city)	146	55.7
Out of Addis Ababa	116	44.3

### Perceived risk of COVID-19

Just over a third (101, 38.5%) of the respondents reported that they had a chronic illness; of whom a third were diabetic (35, 34.6%). Only a quarter of the respondents perceived their level of risk of contracting COVID-19 was “high”. Table 2.

**Table 2:** Perceived risk of COVID-19 among respondents.

Characteristics	Frequency	Percentage
**Do you have a chronic illness? (n = 262)**		
Yes	101	38.5
No	155	59.2
I do not know	6	2.3
**What is your chronic illness? (n = 101)**		
Heart disease	11	10.9
Hypertension	30	29.7
Diabetes	35	34.6
Other*	25	24.8
**High-risk family member (elderly, with chronic illness) (n = 262)**		
Yes	81	30.9
No	181	69.1
**Perceived level of risk for contracting COVID 19 (n = 262)**		
High	73	27.9
Medium	133	50.8
Low	52	19.8
I do not know	4	1.5

*asthma, epilepsy, kidney disease and cancer

### Knowledge of COVID-19 transmission and prevention

Mass media, social media and health professionals were the commonest sources of COVID-19 information. Close to a third of the respondents (82, 31.3%) reported that consuming homemade herbs and spices such as ginger was protective of COVID-19. Overall, just over half (139, 53.0%) of the respondents had adequate knowledge of mode of transmission, symptoms, and prevention methods of COVID-19. Table 3.

**Table 3:** Knowledge of COVID-19 prevention, symptoms, and transmission among respondents.

Characteristics	Frequency	Percentage
**Primary source of information about COVID-19**		
TV or radio	246	93.9
Health professional	141	53.8
Social media	122	46.6
Family or friends	77	29.4
Other (newspaper, religion institutions)	6	2.3
**Ways of COVID-19 transmission**		
Respiratory droplets of infected person	206	78.6
Direct contact with infected person	250	95.4
Indirect contact through infected surface	12	4.6
**Main symptoms of COVID-19**		
Fever	213	81.3
Fatigue	115	43.9
Dry cough	230	87.8
Sore throat	126	48.1
Headache	29	11
Other*	39	14.8
**Modes of prevention of COVID-19**		
Avoid crowded places	97	37
Keep physical distance to a minimum of 2 meters	148	56.5
Wear a mask	242	92.4
Avoid shaking hands	190	72.5
Wash hands with water and soap for at least 20 seconds	191	72.9
Clean hands with alcohol-based sanitizer	165	63
Cover mouth and nose when coughing or sneezing	65	24.8
Avoid touching eyes, nose, or mouth with unwashed hands	65	24.8
Clean repeatedly touched surfaces	26	9.9
Other**	8	3.05
Consuming herbs and spices such as ginger is protective of COVID-19	82	31.3
An infected person cannot transmit the disease if she/he has no cough	26	9.9
No need for a suspected person to be quarantined	9	3.4
**Overall level of knowledge**		
Adequate	139	53
Inadequate	123	47

*chills, shortness of breath, joint pain and sneezing

**drink hot beverages, wear gloves, consume a balanced diet and get vaccinated

### Attitude towards COVID-19 prevention and control measures

Less than half (115, 43.9%) of the respondents felt they had a role in reducing the spread of COVID-19. Overall, the majority (207, 79%) of the study participants had a favorable attitude towards control and prevention measures of the disease. Table 4.

**Table 4:** Attitude of respondents towards COVID-19 prevention and control measures.

Characteristic	Frequency	Percentage
**If I were infected with COVID-19, I would self-isolate**		
Strongly agree	134	51.1
Agree	119	45.4
Neutral	8	3.1
Disagree	0	0
Strongly disagree	1	0.4
**I am willing to get tested for COVID 19**		
Strongly agree	115	43.9
Agree	90	34.4
Neutral	20	7.6
Disagree	26	9.9
Strongly disagree	11	4.2
**I have a role in reducing the spread of COVID-19**		
Strongly agree	126	48.1
Agree	104	39.7
Neutral	20	7.6
Disagree	12	4.6
Strongly disagree	0	0
**If I were infected with COVID-19, I would disclose my status to relatives, friends or colleagues**		
Strongly agree	117	44.7
Agree	104	39.7
Neutral	20	7.6
Disagree	10	3.8
Strongly disagree	11	4.2
**Whether young or old, everyone should equally practice COVID-19 precautions**		
Strongly agree	162	61.8
Agree	86	32.8
Neutral	5	1.9
Disagree	9	3.4
Strongly disagree	0	0
**Overall level of attitude**		
Favorable	207	79
Unfavorable	55	21

### COVID-19 precautionary practices

Above two-third (190, 72.5%) of the respondents always wore face masks when going outside of their house. Overall, just above half (116, 55.7%) of the respondents had a satisfactory practice of taking COVID-19 precautions. Table 5.

**Table 5:** COVID-19 precautionary practices among respondents in the preceding two weeks.

Characteristics	Frequency	Percentage
**Avoided going to crowded places or social gatherings**		
Always	64	24.4
Occasionally	151	57.6
Never	47	17.9
**Wore facemask when leaving home**		
Always	190	72.5
Occasionally	70	26.7
Never	2	0.8
**Avoided shaking hands with other people**		
Always	119	45.4
Occasionally	117	44.7
Never	26	9.9
**Washed hands with water and soap for at least 20 seconds**		
Always	113	43.1
Occasionally	121	46.2
Never	28	10.7
**Kept physical distance to a minimum of 2 meters**		
Always	30	11.5
Occasionally	134	51.1
Never	98	37.4
**Avoided touching eyes, nose or mouth with unclean hands**		
Always	74	28.3
Occasionally	147	56.1
Never	41	15.6
**Covered mouth and nose when coughing or sneezing**		
Always	118	45
Occasionally	113	43.1
Never	31	11.8
**Cleaned hands with alcohol-based sanitizer**		
Always	93	35.5
Occasionally	132	50.4
Never	37	14.1
**Disinfected mobile phone or personal articles with sanitizer**		
Always	68	26
Occasionally	99	37.8
Never	95	36.2
**Overall practice of COVID-19 precautions**		
Satisfactory	116	55.7
Unsatisfactory	146	44.3

### Factors associated with COVID-19 prevention practices

Of the variables tested in the initial binary logistic regression analysis, marital status, perceived risk of contracting COVID-19 and having a high risk family member were not significantly associated with COVID-19 precautionary behaviors. Educational status and knowledge of COVID-19 were associated with the practice of COVID-19 precaution. However, these associations were not significant in the multivariable analysis. In the final multivariable logistic regression model, only two variables particularly the place of residence (Addis Ababa) [AOR: 2.25, 95% CI (1.22–4.15)], and favorable attitude towards COVID-19 prevention and control measures prevention [AOR: 4.88, 95% CI (2.08–11.68)] were significantly associated with practice. Table 6.

**Table 6:** Factors associated with COVID-19 precautionary practices in regression analysis.

Characteristics	Practice		COR (95% CI)	AOR (95% CI)	P-value
	Satisfactory	Unsatisfactory			
**Educational Status**					
No formal education	18(62.1)	11(37.9)	1	1	
Primary	15(42.9)	20(57.1)	0.46(0.17–1.25)	0.33(0.09–1.22)	0.097
Secondary	31(34.4)	59(65.6)	0.32(0.14–0.76)	0.40(0.12–1.29)	0.125
College and above	52(48.1)	56(51.9)	0.57(0.25–1.31)	0.40(0.13–1.29)	0.126
**Place of residence**					
Addis Ababa	81(55.5)	65(44.5)	2.88(1.73–4.82)	2.25(1.22–4.15)*	0.009
Out of Addis Ababa	35(30.2)	81(69.8)	1		
**Knowledge of COVID-19 mode of transmission, symptoms and prevention**					
Satisfactory	72(51.8)	67(48.2)	1.93(1.17–3.17)	1.14(0.6–2.16)	0.697
Unsatisfactory	44(35.8)	79(64.2)	1	1	
**Attitude towards COVID-19 prevention and control measures**					
Favorable	108 (52.8)	99 (47.8)	6.4(2.88–14.23)	4.88(2.08–11.68)*	0.001
Unfavorable	8 (14.5)	47 (85.5)	1		

## Discussion

Just above half (55.7%) of the study participants had a satisfactory level of COVID-19 precautionary practices. The face mask was the most commonly utilized precautionary method. More than two-thirds of the respondents reported they always wore a face mask. This finding was consistent with the knowledge assessment result where almost all of the respondents identified face masks as the primary means of COVID-19 prevention. Moreover, this result is in agreement with previous studies that reported a positive association between COVID-19 knowledge and utilization of face masks [16]. However, according to WHO, the use of a mask alone is insufficient to provide an adequate level of protection and must be combined with hand hygiene to prevent transmission of COVID-19 [17]. In the present study, hand hygiene practice was low, as less than half of respondents reported washing hands with water and soap, and only a third reported using alcohol-based hand sanitizers. Furthermore, the findings in the current study show a decline in hand hygiene practice levels as compared to reports from similar studies conducted a year ago at the start of the pandemic [13, 18]. This finding might indicate inadequate hand hygiene practice might be contributing to hindering the effort to slow the spread of the virus in the study area.

In this study, the lowest score on COVID-19 precautionary practices was that of social distancing; only a quarter of the respondents avoided crowded places or social gatherings, and a mere one-tenth maintained adequate physical distance. This finding also indicates a decline as compared to reports from previous studies [19]. A multitude of factors can explain the poor adherence to physical distancing practice. For one, studies show that the odds of not adhering to all social distancing rules increases if a participant does not self-identify as highly vulnerable to contracting COVID-19 [18, 19]. Congruently, in this study, only a third of the respondents reported a high perceived risk of infection indicating a lower perceived vulnerability. Another possible explanation for a lower adherence to physical distancing could be that maintaining distance may not be always entirely possible due to living in crowded areas [19] and the use of crowded public transportation, leading to unintentional non-adherence to physical distancing guidelines [20]. Furthermore, only a quarter of the study participants in this study avoided going to social gatherings such as weddings, funerals or religious ceremonies. This finding might highlight the possible influence of socio-cultural factors on adherence to COVID-19 precautionary behaviors.

The level of knowledge of respondents on COVID-19 in this study was relatively higher than those findings reported by similar studies [13, 18, 19]. Nonetheless, only half of those surveyed in this study had adequate knowledge—which is unacceptably low. A quarter of the respondents were unable to mention key COVID-19 precautionary methods such as covering the mouth and nose while coughing or sneezing. Furthermore, close to a third of the respondents agreed with the statement that consuming herbs and spices were protective against COVID-19. In addition, close to a quarter of respondents believed that an infected person cannot transmit the disease if she/he has no cough. These findings are contrary to expectations as public mainstream media with a widespread outreach consistently provide accurate COVID-19 related information. Studies indicate that communicable diseases and vaccination are one of the domains of health misinformation [21, 22]. In the current study, the observed health misinformation and myths might be attributed to the growing utilization of social media. Almost half of the participants in this study reported utilizing social media as a source of health information. However, the evidence in this study is insufficient, and further exploration is needed on the influence of sources and quality of COVID-19 information on the adoption of COVID-19 precautionary practices.

The KAP (Knowledge, Attitude, and Practices) theory states that for the adoption and formation of new behavior, both the acquisition of knowledge and generation of attitude is important [23]. In this study, a favorable attitude towards COVID-19 preventive measures was strongly associated with good practice of personal precautions. Likewise, previous studies have reported positive attitude has a significant and robust impact on the practice of disease preventive behavior [6, 19, 24, 25]. For instance, participants who felt COVID-19 will be successfully controlled were more likely to refrain from handshaking [13]. Similarly, a favorable attitude predicted the intention of handwashing, one of the key precautionary measures against COVID-19 [24]. This finding might have implications for the adoption of public health education strategies targeted at improving attitude towards COVID-19 control measures among a segment of the community with poor practice.

In this study, as compared to being from outside the capital city, being a resident of the capital city, Addis Ababa, was associated with better COVID-19 precautionary practices. The possible explanation for this could be the relatively strict enforcement of COVID-19 Public Health and Social Measures (PHSM) in the capital city as compared to other parts of the country [4]. For instance, unlike other cities or towns of the country, a face mask is required to receive any public service in the capital city. This finding was consistent with studies that reported implementation of mask mandates led to widespread uptake of masks [26, 27]. This result highlights the possible implication of strong regulations on improving public compliance with COVID-19 precautionary measures.

This study has several limitations. The population studied was clients attending a hospital and might not be representative of the general public. This might have overestimated the reported level of certain factors such as perceived risk of COVID-19, which might be higher among clients seeking health care. The study assessed COVID-19 precautionary practices based on self-report, which might have inflated the true magnitude of proper practices among the respondents. Due to the study design implemented, it is not possible to assess whether some risk factors modified COVID-19 precautionary practices or changed as a result of practice.

## Conclusions

The level of COVID-19 precautionary practices among clients vising the tertiary center was unsatisfactory. Having a favorable attitude towards COVID-19 prevention measures had a strong association with satisfactory COVID-19 precautionary practices. Stricter public enforcement of COVID-19 preventive measures might have contributed to better practice of precaution. Public health education strategies targeted at improving attitude by communicating the effectiveness of proper practices for the prevention of COVID-19 spread might be beneficial.

## Supporting information

S1 File Survey questionnaire in English and Amharic. (DOC)

## Abbreviations

AOR: Adjusted Odds Ratio
CI: Confidence Interval
COVID-19: Coronavirus Disease
COR: Crude Odd Ratio
PHSM: Public Health and Social Measures
SARS-CoV-2: Severe Acute Respiratory Syndrome Coronavirus 2
