# Supplementary material for: COVID-19 precautionary practices and associated factors among clients visiting a tertiary hospital, Addis Ababa, Ethiopia
Source: PLoS One. 2022 Apr 18;17(4):e0267000. doi: 10.1371/journal.pone.0267000 (PMC9015144; doi:10.1371/journal.pone.0267000)
Supplement: S1 File — (DOC) [file pone.0267000.s001.doc]

**Additional file 1 - Data collection tool in English**

| **Part one: Socio-demographic status** | | | |
| --- | --- | --- | --- |
| **S. No** | **Questions** | **Option/Response** | **Skip** |
| 101 | Age (in years) |  |  |
| 102 | Sex | 1. Male 2. Female |  |
| 103 | Ethnicity | 1. Tigre 2. Amhara 3. Oromo 4. Others (Specify) ___________ |  |
| 104 | Religion | 1. Orthodox 2. Muslim 3. Protestant 4. Catholic 5. Others (Specify)____________ |  |
| 105 | Marital Status | 1. Single 2. Married 3. Divorced 4. Widowed 5. Separated |  |
| 106 | Educational status | 1. Unable to read and write 2. Able to read and write 3. Primary /1-8th grade/ 4. Secondary /9-12th grade/ 5. College and above |  |
| 107 | Occupational status | 1. Government employed 2. Daily laborer 3. House wife 4. Merchant 5. Private business/employed 6. Student 7. Currently unemployed 8. Others (specify)________ |  |
| 108 | Place of residence | 1. Addis Ababa  2. Out of Addis Ababa |  |
| 109 | Estimated monthly income | _________ETB |  |
| 110 | How many people live in your household, including yourself? |  |  |
| **Part two: Perceived risks of COVID-19** | | | |
| 201 | Are there high risk individuals from your family member? (elderly or with chronic illness)? | 1. Yes 2. No |  |
| 202 | Do you have a chronic illness? | 1. Yes 2. No | If No, skip to Qn. 204 |
| 203 | What is your chronic illness? | 1. Heart disease 2. Hypertension 3. Diabetes 4. Asthma 5. Cancer 6. HIV 7. Other (Specify)_______ |  |
| 204 | How do you rate your level of risk for contracting COVID 19? | 1. High 2. Medium 3. Low 4. I do not know |  |
| **Part three: Knowledge of COVID-19** | | | |
| 301 | How is COVID-19 transmitted? **(Multiple response possible)**  **DO NOT READ OPTIONS!** | 1. Respiratory droplets of infected person 2. Direct contact with infected person 3. Indirect contact (with infected surface) 4. Other (Specify)________ |  |
| 302 | What are the main symptoms of COVID-19? **(Multiple response possible)**  **DO NOT READ OPTIONS!** | 1. Fever 2. Fatigue 3. Dry cough 4. Sore throat 5. Headache 6. No symptoms 7. Other (Specify)________ |  |
| 303 | What are modes of prevention of COVID-19? **(Multiple response possible)**  **DO NOT READ OPTIONS!** | 1. Avoid crowded places 2. Keep physical distance to a minimum of 2 meters 3. Wear a mask 4. Stop shaking hands 5. Wash hands with water & soap for at least 20 seconds 6. Clean hands with alcohol-rubbing or sanitizer 7. Cover mouth/nose with elbow when coughing/sneezing 8. Not touch eyes, nose or mouth with unwashed hands 9. Other (specify)_________ |  |
| 304 | Eating lemon, *feto* or other spices can protect from COVID-19) | 1. Correct  2. Incorrect  3. I do not know |  |
| 305 | A person who had contact with someone infected or suspected with the COVID-19 virus should be quarantined or isolated. | 1. Correct 2. Incorrect 3. I do not know |  |
| 306 | A person infected with COVID cannot transmit the disease if he has no cough. | 1. Correct 2. Incorrect 3. I do not know |  |
| 307 | What is your primary source of information about Corona? **(Multiple response possible)** | 1. TV/Radio/Newspaper  2. Health professionals  3. Social media  4. Family/Friends  5. Other (specify)_________ |  |
| **Part four: COVID-19 preventive practices in the last two weeks** | | | |
| 401 | Within the last two weeks, have you refrained from going to crowded places or social gatherings? | 1. Always 2. Occasionally 3. Never |  |
| 402 | Within the last two weeks, have you worn a mask when leaving home? | 1. Always 2. Occasionally 3. Never |  |
| 403 | Within the last two weeks, have you refrained from shaking hands with other people? | 1. Always 2. Occasionally 3. Never |  |
| 404 | Within the last two weeks, have you been washing your hands with water & soap for at least 20 seconds? | 1. Always 2. Occasionally 3. Never |  |
| 405 | Within the last two weeks, have you kept physical distance to a minimum of 2 meters between you and other persons? | 1. Always 2. Occasionally 3. Never |  |
| 406 | Within the last two weeks, have you avoided touching your eyes, nose or mouth before washing hands? | 1. Always 2. Occasionally 3. Never |  |
| 407 | Within the last two weeks, have you covered your mouth/nose with elbow when coughing/sneezing? | 1. Always 2. Occasionally 3. Never |  |
| 408 | Within the last two weeks, have you frequently cleaned your hands with sanitizer? | 1. Always 2. Occasionally 3. Never |  |
| 409 | Within the last two weeks, have you frequently disinfected your mobile phone or personal articles? | 1. Always 2. Occasionally 3. Never |  |
| 410 | Is the person wearing a mask now?  (observe and fill in) | 1. Yes 2. No |  |
| **Part-five: Attitude towards COVID-19** | | | |
| 501 | If I were infected with COVID-19, I would self-isolate | 1. Strongly agree 2. Agree 3. Neutral 4. Disagree 5. Strongly disagree |  |
| 502 | I am willing to do a voluntary test for COVID 19 | 1. Strongly agree 2. Agree 3. Neutral 4. Disagree 5. Strongly disagree |  |
| 503 | As an individual, I have a role in stopping the spread of the COVID-19 | 1. Strongly agree 2. Agree 3. Neutral 4. Disagree 5. Strongly disagree |  |
| 504 | If I were infected with COVID-19, I would tell my friends or colleagues | 1. Strongly agree 2. Agree 3. Neutral 4. Disagree 5. Strongly disagree |  |
| 505 | Whether younger or older, everyone should equally implement COVID-19 prevention practice | 1. Strongly agree 2. Agree 3. Neutral 4. Disagree 5. Strongly disagree |  |

**Data collection tool in Amharic**

መለያ ኮድ ቁጥር: _____________________

የሆስፒታሉ ስም፡ _____________________

| **ክፍል 1 ፡ ማህበራዊና ኢኮኖሚያዊ መረጃ** | | | |
| --- | --- | --- | --- |
| **ተ.ቁ.** | **ጥያቄ** | **መልስ** | **ዝለል** |
| **101** | እድሜዎ ስንት ነው? | 1. __________ ዓመት |  |
| **102** | ፆታ | 1. ወንድ  2. ሴት |  |
| **104** | ሐይማኖትዎ ምንድን ነው? | 1. ኦርቶዶክስ 2. ሙስሊም 3. ፕሮቴስታንት 4. ሌላ (ይግለጹት ________________________ |  |
| **105** | የጋብቻ ሁኔታ | 1. ያላገባ 2. ያገባ 3. የፈታ/በሕግ 4. የሞተበት 5. የተለያዩ |  |
| **106** | የትምህርት ደረጃ | 1. ያልተማረ 2. መጻፍና ማንበብ የሚችል 3. የመጀመርያ ደረጃ 4. ሁለተኛ ደረጃ 5. ኮሌጅና ከዛ በላይ |  |
| **107** | የስራ ሁኔታ | 1. የመንግስት ሰራተኛ 2. የቀን ሰራተኛ/የግል ስራ/ተቀጣሪ 3. የቤት እመቤት 4. ነጋዴ 5. ተማሪ 6. ስራ አጥ 7. ሌላ (ይግለጹ)___________ |  |
| **108** | የመጡበት ቦታ | 1. ከአዲስ አበባ  2. ከአዲስ አበባ ውጪ |  |
| **109** | ወርሐዊ ገቢ | _____________ብር |  |
| **110** | እርስዎን ጨምሮ አብረዎት የሚኖሩት የሰዎች ብዛት ስንት ነው? | _______________ |  |
| **ክፍል 2፡ ለኮሮና ቫይረስ የመጋለጥ ስጋት** | | | |
| **201** | በቤትዎ ከፍተኛ የጤና ስጋት ያለበት የቤተሰብ አባል አለ? (ለምሳሌ፦ በዕድሜ የገፉ እና ተላላፊ ያልሆነ ፥ ሥር የሰደደ (**Chronic**) በሽታ) | 1. አዎ 2. የለም |  |
| **202** | እርስዎ ሥር የሰደደ (**Chronic**)በሽታ አለብዎት? (ለምሳሌ: የደም ግፊት ፣ የስኩዋር በሽታ…) | 1. አዎ 2. የለም 3. አላውቅም | ‘የለም’ ወይም  ‘አላውቅም'ካሉ ወደ ጥያቄ **204** ይሂዱ |
| **203** | ያለብዎት ሥር የሰደደ (**Chronic**) በሽታ ምንድን ነው? | 1. የልብ በሽታ 2. የደም ግፊት 3. የስኩዋር በሽታ 4. ካንሰር 5. ኤች አይ ቪ 6. ሌላ (ይግለጹ)_________ |  |
| **204** | ለኮሮና ቫይረስ ያለዎትን ተጋላጭነት እንዴት ይመዝኑታል? | 1. ከፍተኛ 2. መካከለኛ 3. ዝቅተኛ 4. አላውቅም |  |
| **ክፍል 3: ስለ ኮሮና ቫይረስ በሽታ እውቀት** | | | |
| **301** | የኮሮና ቫይረስ በሽታ መተላለፊያ መንገዶች ምን ምንድን ናቸው?    **ምርጫዎቹን አያንብቡላቸው!!**  **(ከ አንድ በላይ መልስ መምረጥ ይቻላል)** | 1. በቫይረሱ በተያዙ ግለሰቦች ትንፋሽ ጠብታዎች (Droplet) በኩል 2. በቫይረሱ በተያዙ ግለሰቦች ጋር የሚደረግ ንክኪ (ምሳሌ: መጨባበጥ) 3. በተበከለ ምግብ/ውሀ 4. ሌላ (ይግለጹ)____________ |  |
| **302** | የ የኮሮና ቫይረስ በሽታ ዋና ዋና ምልክቶች ምን ምን ናቸው?  **ምርጫዎቹን አያንብቡላቸው!!**  **(ከ አንድ በላይ መልስ መምረጥ ይቻላል)** | 1. ትኩሳት 2. ድካም 3. ደረቅ ሳል 4. የጉሮሮ መከርከር 5. ምልክት የለውም 6. ሌላ (ይግለጹ)_________ |  |
| **303** | የኮሮና ቫይረስ በሽታ መከላከያ መንገዶች ምን ምን ናቸው?  **ምርጫዎቹን አያንብቡላቸው!!**  **(ከ አንድ በላይ መልስ መምረጥ ይቻላል)** | 1. በተጨናነቁ ስፍራዎች እና የህዝብ መጓጓዣዎችን አለመጠቀም 2. 2 ሜትር አካላዊ ርቀት መጠበቅ 3. የአፍ እና አፍንጫ ጭንብል መጠቀም 4. አለመጨባበጥ 5. እጅን በውኃ እና በሳሙና ለ20 ሰኮንድ መታጠብ 6. እጅን በአልኮል/በሳኒታይዘር ማፅዳት 7. ለማሳል/ለማስነጠስ ክንድን ሽፋን መጠቀም 8. ባልታጠበ እጅ ዓይን ፣ አፍንጫ ፣ አፍን አለመንካት 9. በተደጋጋሚ የሚነኩ (ምሳሌ፣ የበር እጄታዎች…) ማጽዳት 10. ሌላ (ይግለጹ)_________ |  |
| **304** | ዝንጅብል ወይም ፌጦ መጠቀም የኮሮና ቫይረስ በሽታን ይከላከላል:: | 1. ትክክል ነው 2. ትክክል አይደለም 3. አላውቅም |  |
| **305** | በ ኮሮና ቫይረስ ከተያዘ ሰው ጋር ግንኙነት ያላቸው ሰዎች እራሳቸውን መለየት (በለይቶ ማቆያ መግባት) አለባቸው፡፡ | 1. ትክክል ነው 2. ትክክል አይደለም 3. አላውቅም |  |
| **306** | በ ኮሮና ቫይረስ የተያዘ ሰው የማያስለው ከሆነ በሽታውን አያስተላልፍም። | 1. ትክክል ነው 2. ትክክል አይደለም 3. አላውቅም |  |
| **307** | ስለ ኮሮና ቫይረስ መረጃ በዋነኝነት ከየት ነው ያገኙት?  **ምርጫዎቹን አያንብቡላቸው!!**  **(ከ አንድ በላይ መልስ መምረጥ ይቻላል)** | 1. ቲቪ/ሬድዮ/ጋዜጣ 2. የጤና ባለሙያ 3. ማህበራዊ ሚዲያ (ፌስ ቡክ…) 4. ቤተሰብ/ጉዋደኛ 5. ሌላ (ይግለጹት)_________ |  |
| **ክፍል-4: የኮሮና** **በሽታን የመከላከል ልምድ** | | | |
| **401** | ባለፉት ሁለት ሳምንታት ውስጥ **ወደ ተጨናነቀ ስፍራ ከመሄድ ይልቅ በቤት ቆይተዋል?** | 1. ሁልጊዜ 2. አልፎ አልፎ 3. በፍጹም |  |
| **402** | ባለፉት ሁለት ሳምንታት ውስጥ **ከቤት ሲወጡ የአፍ እና አፍንጫ ጭምብል ይለብሱ ነበር?** | 1. ሁልጊዜ 2. አልፎ አልፎ 3. በፍጹም |  |
| **403** | ባለፉት ሁለት ሳምንታት ውስጥ **ለሰላምታ እጅ ከመጨበጥ ይቆጠቡ ነበር?** | 1. ሁልጊዜ 2. አልፎ አልፎ 3. በፍጹም |  |
| **404** | ባለፉት ሁለት ሳምንታት ውስጥ **እጅዎትን በውኃ እና በሳሙና ለ20 ሰኮንድ ይታጠቡ ነበር?** | 1. ሁልጊዜ 2. አልፎ አልፎ 3. በፍጹም |  |
| **405** | ባለፉት ሁለት ሳምንታት ውስጥ **አካላዊ ርቀትን በ2 ሜትር ይጠብቁ ነበር?** | 1. ሁልጊዜ 2. አልፎ አልፎ 3. በፍጹም |  |
| **406** | ባለፉት ሁለት ሳምንታት ውስጥ **እጅዎን ሳይታጠቡ ዓይን ፣ አፍንጫ ፣ አፍን ከመንካት ይቆጠቡ ነበር?** | 1. ሁልጊዜ 2. አልፎ አልፎ 3. በፍጹም |  |
| **407** | ባለፉት ሁለት ሳምንታት ውስጥ **ለማሳል/ለማስነጠስ ክንድዎን ለሽፋን ይጠቀሙ ነበር?** | 1. ሁልጊዜ 2. አልፎ አልፎ 3. በፍጹም |  |
| **408** | ባለፉት ሁለት ሳምንታት ውስጥ **እጅዎን በአልኮል/በሳኒታይዘር ያጸዱ ነበር?** | 1. ሁልጊዜ 2. አልፎ አልፎ 3. በፍጹም |  |
| **409** | ባለፉት ሁለት ሳምንታት ውስጥ **ሞባይሎትን ወይም የግል መጠቀሚያ እቃዎችን በአልኮል/በሳኒታይዘር ያጸዱ ነበር?** | 1. ሁልጊዜ 2. አልፎ አልፎ 3. በፍጹም |  |
| **410** | አሁን ተጠያቂው **የአፍ እና አፍንጫ ጭንብል አድርገዋል?**  **(አይተው ይሙሉ)** | 1. አዎ 2. አላደረጉም |  |
| **ክፍል-5: ስለ ኮሮና** **በሽታ አመለካከት** | | | |
| **501** | የ ኮሮና ቫይረስ ቢገኝብኝ እራሴን እለያለሁ | 1. በጣም እስማማለሁ 2. እስማማለሁ 3. አልቃወምም አልስማማም 4. አልስማማም 5. በጣም አልስማማም |  |
| **502** | የ ኮሮና ቫይረስ ምርመራ ለማድረግ ፈቃደኛ ነኝ። | 1. በጣም እስማማለሁ 2. እስማማለሁ 3. አልቃወምም አልስማማም 4. አልስማማም 5. በጣም አልስማማም |  |
| **503** | የ ኮሮና ቫይረስ ቢገኝብኝ ለማውቃቸው ሰዎች/የስራ ባልደረቦች አሳውቃለሁ። | 1. በጣም እስማማለሁ 2. እስማማለሁ 3. አልቃወምም አልስማማም 4. አልስማማም 5. በጣም አልስማማም |  |
| **504** | እንደ ግለሰብ የኮሮና ቫይረስን ስርጭት ለመቀነስ ሚና አለኝ። | 1. በጣም እስማማለሁ 2. እስማማለሁ 3. አልቃወምም አልስማማም 4. አልስማማም 5. በጣም አልስማማም |  |
| **505** | በየትኘውም የእድሜ ክልል ላይ ያለ ሰው ለኮሮና ቫይረስ ጥንቃቄ ማድረግ አለበት። | 1. በጣም እስማማለሁ 2. እስማማለሁ 3. አልቃወምም አልስማማም 4. አልስማማም 5. በጣም አልስማማም |  |
